# Myocyte enhancer factor 2D provides a cross-talk between chronic inflammation and lung cancer

**DOI:** 10.1186/s12967-017-1168-x

**Published:** 2017-03-24

**Authors:** Hai-xing Zhu, Lin Shi, Yong Zhang, Yi-chun Zhu, Chun-xue Bai, Xiang-dong Wang, Jie-bai Zhou

**Affiliations:** 1 Department of Pulmonary Medicine, Ruijin Hospital, Shanghai Jiaotong University School of Medicine, Shanghai, China; 2 Department of Pulmonary Medicine, Zhongshan Hospital, Fudan University, 180 Fenglin Road, Shanghai, 200032 China; 3 Shanghai Respiratory Research Institute, Shanghai, China; 4 Institute of Clinical Science, Zhongshan Hospital, Fudan University, Shanghai, China; 5 Fudan University Center for Clinical Bioinformatics, Shanghai, China

**Keywords:** Clinical bioinformatics, Inflammation, Lung cancer, Biomarker, Myocyte enhancer factor 2D

## Abstract

**Background:**

Lung cancer is the leading cause of cancer-related morbidity and mortality worldwide. Patients with chronic respiratory diseases, such as chronic obstructive pulmonary disease (COPD), are exposed to a higher risk of developing lung cancer. Chronic inflammation may play an important role in the lung carcinogenesis among those patients. The present study aimed at identifying candidate biomarker predicting lung cancer risk among patients with chronic respiratory diseases.

**Methods:**

We applied clinical bioinformatics tools to analyze different gene profile datasets with a special focus on screening the potential biomarker during chronic inflammation-lung cancer transition. Then we adopted an in vitro model based on LPS-challenged A549 cells to validate the biomarker through RNA-sequencing, quantitative real time polymerase chain reaction, and western blot analysis.

**Results:**

Bioinformatics analyses of the 16 enrolled GSE datasets from Gene Expression Omnibus online database showed myocyte enhancer factor 2D (MEF2D) level significantly increased in COPD patients coexisting non-small-cell lung carcinoma (NSCLC). Inflammation challenge increased MEF2D expression in NSCLC cell line A549, associated with the severity of inflammation. Extracellular signal-regulated protein kinase inhibition could reverse the up-regulation of MEF2D in inflammation-activated A549. MEF2D played a critical role in NSCLC cell bio-behaviors, including proliferation, differentiation, and movement.

**Conclusions:**

Inflammatory conditions led to increased MEF2D expression, which might further contribute to the development of lung cancer through influencing cancer microenvironment and cell bio-behaviors. MEF2D might be a potential biomarker during chronic inflammation-lung cancer transition, predicting the risk of lung cancer among patients with chronic respiratory diseases.

**Electronic supplementary material:**

The online version of this article (doi:10.1186/s12967-017-1168-x) contains supplementary material, which is available to authorized users.

## Background

Lung cancer is the most prevalent malignant tumor and the leading cause of cancer-related morbidity and mortality worldwide [1], mainly cataloged into non-small-cell lung carcinoma (NSCLC), large-cell carcinoma (LCC), and small-cell lung carcinoma (SCLC). Patients with chronic respiratory diseases are predisposed to higher incidence of lung cancer [2–7]. Chronic obstructive pulmonary disease (COPD), a chronic inflammatory lung disease, is closely related to susceptibility to lung cancer and stands as the most important risk factor of lung cancer among smokers [8]. Patients with moderate-to-severe COPD have a fivefold higher risk of developing lung cancer than smokers without the disease [9]. Chronic inflammation associated with COPD seems to play a critical role in cancer evolution. However, there is limited knowledge how the transition from chronic inflammation to lung cancer is triggered. The emergency of genomics and clinical bioinformatics allows defining gene alterations during the process of carcinogenesis to further understand the relationship between chronic inflammation and the onset of lung cancer.

The myocyte enhancer factor 2 (MEF2) family of human transcription factors, consisting of four subtypes, MEF2-A, -B, -C and -D, have a diversity of functions in different tissues and have been implicated in numerous diseases. MEF2s play an important role in the activation of the genetic processes that control cell differentiation, proliferation, and apoptosis in a wide range of cell types [10]. It has been reported that MEF2 is correlated with lower physical activity in COPD patients [11]. Altered MEF2 activity has been revealed as driver of cancer development, including both hematological cancers and solid tumor. Recent study found that MEF2 genes might act as oncogenes in NSCLC [12].

The present study is aimed to figure out candidate “communicator” between chronic inflammation and lung cancer and determine the potential regulatory mechanism. We highlight studies exploring the link between inflammation and cancer and discuss emerging biomarker for predicting risk of lung cancer among patients with chronic respiratory diseases.

## Methods

### Bioinformatics tools

We used “lung cancer” as the search keyword and “human” as organism to search for GSE datasets in Gene Expression Omnibus (GEO) online database. GSE datasets containing comparative gene expression profiling between lung cancer and non-cancer tissues, pathological definitions, and information of data normalizing methods were enrolled in the present study. Gene expression profiling data of each dataset were restored to original data, re-normalized with reference genes, and transformed to the ratio of expression data, and then integrated into one dataset. Genes with twofolds and more than twofolds up or down change in lung cancer subtypes were thought to be significantly differential genes, compared with non-cancer tissues. All the differential genes were used in further bioinformatics analysis.

### Cell culture

Human NSCLC cell line A549 cells, human bronchial epithelial cell line HBE cells, and human normal bronchial epithelial cell line BEAS-2B cells were obtained from Shanghai Institute for Biological Science. Cells were cultured in DMEM (high glucose, Hyclone, Logan, UT, USA), supplemented with 10% FBS (Hyclone, Logan, UT, USA), 100 U/ml penicillin, and 100 μg/ml streptomycin at 37 °C in a 5% CO_2_, 95% air environment in humidified incubators.

### Ion Torrent-based RNA-sequencing

RNA extraction was performed using the TRIZOL™LS reagent (Invitrogen, Carlsbad, CA, USA). rRNA depletion was performed using the RiboMinus™ Eukaryote System v2 (Ambion^®^) following standard protocols. First, total RNA was hybridized with biotinylated RiboMinus™ Eukaryote Probe Mix v2. Next, the rRNA-probe complexes were removed from the total RNA by captured with streptavidin-conjugated RiboMinus™ Magnetic Beads. The resulting rRNA-depleted RNA was concentrated and purified with Nucleic Acid Binding Beads. The cDNA libraries were barcoded using the ion total RNA-Seq Kit v2 (Ambion^®^) following standard protocols. RNA sequencing was performed on the Ion Proton™ System (Life Technologies, Carlsbad, CA, USA). Data analysis was performed using Torrent Suite™ Software 4.0 (Ion Torrent). Genes with twofolds and more than twofolds up or down change in LPS-stimulated A549 cells were thought to be significant, compared with the control group.

### Quantitative real time polymerase chain reaction (qRT-PCR) analysis

RNA extraction was performed using the TRIZOL™LS reagent (Invitrogen, Carlsbad, CA, USA). cDNA was prepared using PrimeScript^®^ RT reagent Kit (Takara, Shiga, Japan) following standard protocols. qRT-PCR was performed using SYBR^®^ Premix Ex Taq™ (Takara, Shiga, Japan) on the ABI 7000 PCR instrument (Eppendorf, Hamburg, Germany) with the following two-stage program parameters: 1 min at 95 °C and then 40 cycles of 5 s at 95 °C and 30 s at 60 °C. All samples were run in triplicate, and each group had six wells. Results were shown as relative target mRNA levels. The sequences of the primers used for this analysis are as follows:

MEF2A Forward, 5′-CCGACTGCCTACAACACTGA-3′,

MEF2A Reverse, 5′-GATAACTGCCCTCCAGCAAC-3′;

MEF2B Forward, 5′-AAGTTCGGGCTGATGAAGAA-3′,

MEF2B Reverse, 5′-CATACTGGAAGAGGCGGTTG-3′;

MEF2C Forward, 5′-CTGGCAACAGCAACACCTAC-3′,

MEF2C Reverse, 5′-GAAGGCAGGGAGAGATTTG-3′;

MEF2D Forward, 5′-CACCTGACAATCACCCACAC-3′,

MEF2D Reverse, 5′-AGCATCACCATACAGCACGA-3′;

GAPDH Forward, 5′-CCACCCATGGCAAATTCCATGGCA-3′,

GAPDH Reverse, 5′-TCTACACGGCAGGTCAGGTCCACC-3′.

### Western bolt analysis

Intracellular protein was extracted by radio immunoprecipitation assay lysis buffer. Protein samples (50 μg) were mixed with an equal volume of 5× sodium dodecyl sulfate buffer, boiled for 5 min, and then separated through 10% sodium dodecyl sulfate-polyacrylamide gel electrophoresis gels. Proteins were transferred to polyvinylidene fluoride membranes by electrophoretic transfer after electrophoresis. Membranes were blocked in 5% dry milk for 2 h, rinsed, and incubated with primary antibodies (diluted at their instructions) in TBS thrice at 4 °C overnight. Primary antibody was then removed by washing in TBS and labeled by incubating with 0.1 mg/ml peroxidase-labeled secondary antibodies (against mouse and rabbit) for 2 h. Following three washes in TBS, bands were visualized by ECL (Tanon, Shanghai, China) and exposed to X-ray film. The band densities were quantified with Image J. The results were presented as ratio of band density to total actin.

### Small interfering RNA (siRNA) transfection

Three different sequences targeting MEF2D (Additional file 1: Table S1) were designed and provided by GenePharma (Shanghai, China). siRNA transfection was performed according to the manufacturer’s protocol. Briefly, 1 μl Lipofectamine 2000 and 50 pmol each siRNA were mixed. Cells were then transfected with Lipofectamine/siRNA complexes and incubated at 37 °C for 24 h. Transfection efficiency was evaluated by qRT-PCR and Western Blot analysis. Clones with optimized transfection were selected for stable transfection of MEF2D and used in the experiments.

### Alive measurement of cell bio-behaviors

The cell bio-behaviors including total cell number, cell differentiation, and cell movement were dynamically measured through a Cell-IQ platform (Chip-Man Technologies, Tampere, Finland), equipped with a phase-contrast microscope (Nikon CFI Achromat phase contrast objective with ×10 magnification) and a camera (Nikon, Fukasawa, Japan). The equipment was controlled by Imagen software (Chip-Man Technologies). Images were captured every 5 min for 72 h. Analysis was carried out with a freely distributed Image software (Cell-IQ Imagen v2.9.5c; McMaster Biophotonics Facility, Hamilton, ON, Canada), using the Manual Tracking plug-in created by Fabrice Cordelieres (Institut Curie, Orsay, France). Cell-IQ system can monitor and record time-lapse data through machine vision technology, as well as analyze and quantify cell functions and morphological parameters. Here, we use this system to calculate cell numbers of each stage (dividing/stable stage) during proliferation, discriminate cell stage, and quantify the movement of each individual cell. Each group contained 6–12 replicate image sites.

### Cell migration assay

In the Transwell assay (Corning Inc., Corning, NY, USA), cells were seeded in the upper chamber at the concentration of 5 × 10^5^/ml in serum-free medium, while medium containing 10% FBS was added to the lower chamber. Cells migrated through the permeable membrane at 48 h were fixed and stained with Giemsa, and then counted under microscope.

### Cell proliferation assay

In the cell proliferation assay, cells were seeded in 96-well plates at the concentration of 1 × 10^4^/well and incubated with AR (40 ng/ml) for 24, 48 or 72 h at 37 °C with 5% CO_2_, after which 10 μl of the Cell Counting Kit-8 solution (Dojindo, Japan) was added to the medium. After 2-h incubation, the amount of orange formazan dye generated was determined by measuring the absorbance at 450 nm under microplate reader (Thermo Scientific, Carlsbad, CA, USA).

### Statistical analysis

All values were expressed as mean ± SEM. Statistical analysis was performed using SPSS software (SPSS 20.0; SPSS Inc; Chicago, IL, USA). Values between lung cancer subtypes were analyzed with Students’s *t* test and Mann–Whitney *U* test. Data were evaluated using ANOVA with LSD test for multiple comparisons and Students’s *t* test between two groups. *p* < 0.05 was considered as statistical significant.

## Results

### Elevated MEF2D level in COPD patients with NSCLC

**Fig. 1:**
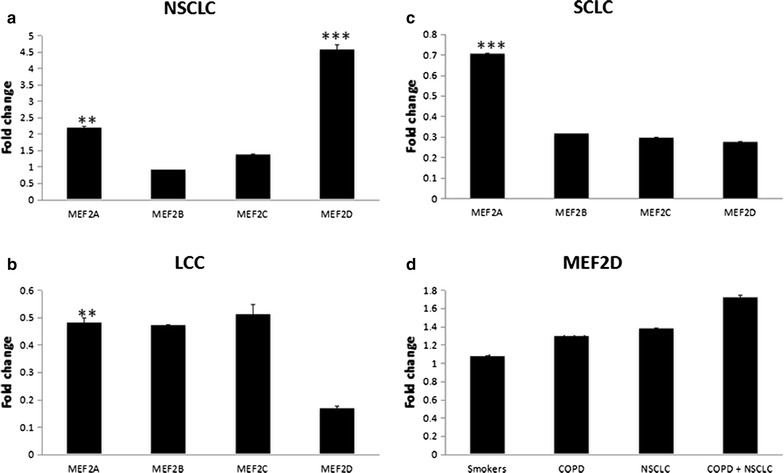
Expressions of MEF2 family genes in patients with lung cancer. The expression of MEF2s in patients with non-small-cell lung carcinoma (NSCLC) (**a**), large-cell carcinoma (LCC) (**b**), small-cell lung carcinoma (SCLC) (**c**). **p < 0.01, and ***p < 0.001, as compared with genes in non-cancer tissues. The expression of MEF2D in smokers, COPD patients, NSCLC patients, and COPD patients coexisting NSCLC (**d**). *p < 0.05, and ***p < 0.001, as compared with genes in healthy non-smokers

16 GSE datasets containing comparative gene expression profiles of lung cancer and non-cancer tissues, pathological definitions, and information of data normalizing methods were enrolled in the present study through clinical bioinformatics tools. There were totally 677 NSCLC, 9 LCC, 56 SCLC cases, and 590 non-cancer cases. In patients with NSCLC, the expressions of MEF2A and MEF2D were significantly higher, compared with non-cancer tissues (Fig. 1a, p < 0.01 and 0.001, respectively). In patients with LCC or SCLC, the expressions of MEF2A were significantly lower, compared with non-cancer tissues (Fig. 1b, c, p < 0.01, and 0.001, respectively). The MEF2D levels were also decreased in patients with LCC or SCLC as compared with non-cancer tissues (Fig. 1b, c). Further data mining demonstrated significantly higher MEF2D level in COPD patients with NSCLC than that in smokers, patients with COPD and no NSCLC, or patients with NSCLC and no COPD, compared with healthy non-smokers (Fig. 1d, p < 0.001).

### Increased MEF2D expression inflammation-activated NSCLC cell line

**Fig. 2:**
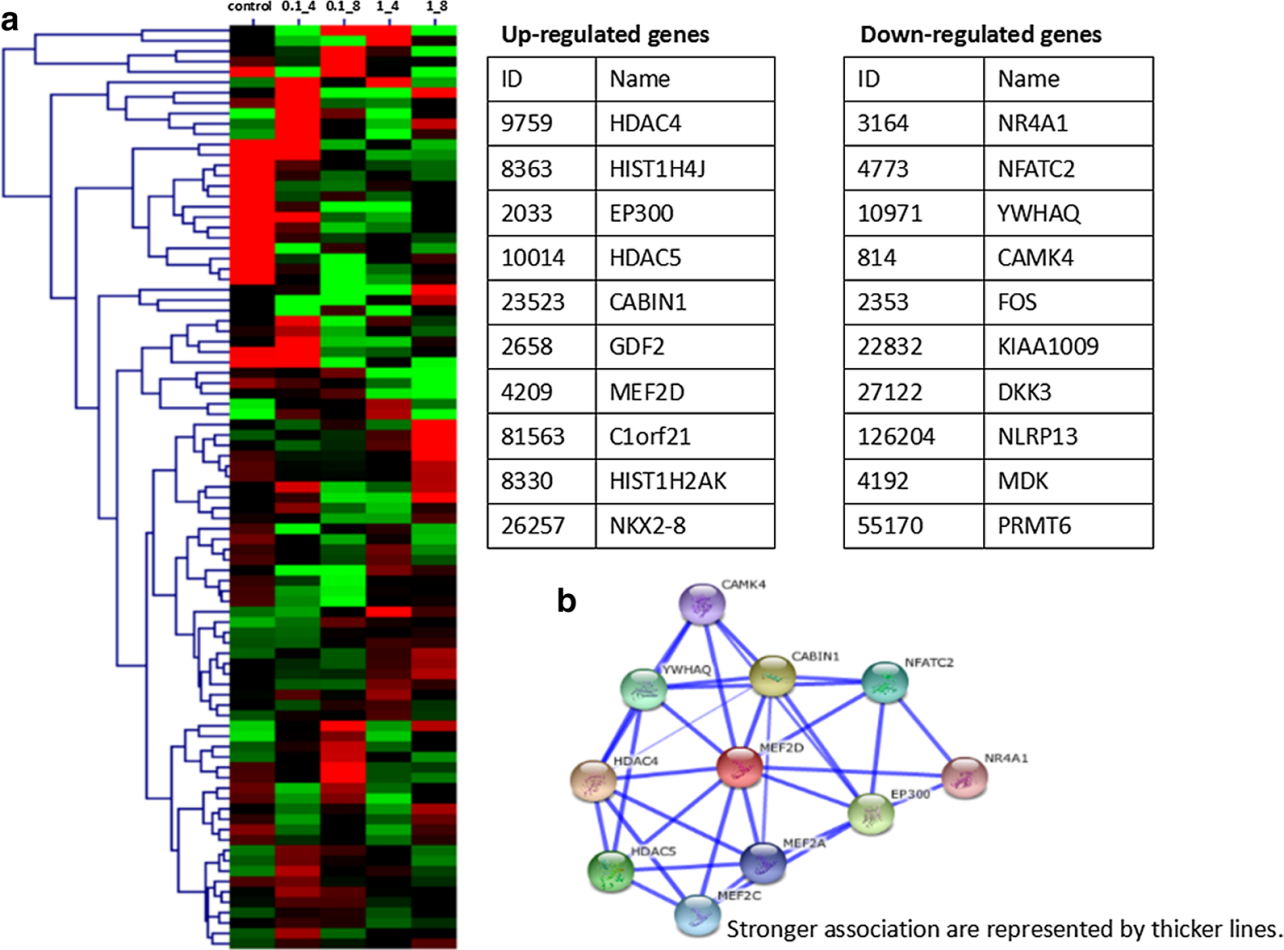
Alterations of gene clusters in inflammation-activated NSCLC cell line. **a** The top ten genes expression more than twofold up- or down-expressed in LPS-stimulated A549 cells; **b** genes associated with MEF2D network

We carried out Ion Torrent-based RNA-sequencing in a model of LPS-challenged NSCLC cell line A549. A549 cells were stimulated with vehicles or LPS at 0.1, 1 μg/ml for 4 h or 8 h, respectively. Figure 2a demonstrated the top ten genes up-expressed over twofold: HDAC4, HIST1H4J, EP300, HDAC5, CABIN1, GDF2, MEF2D, C1orf21, HIST1H2AK, and NKX2-8, as well as the top ten genes down-expressed over twofold: NR4A1, NFATC2, YWHAQ, CAMK4, FOS, KIAA1009, DKK3, NLRP13, MDK, and PRMT6, in LPS-stimulated A549 cells. Figure 2b showed the gene network associated with MEF2D.

### MEF2D specifically up-regulated in inflammation-activated NSCLC cell line

**Fig. 3:**
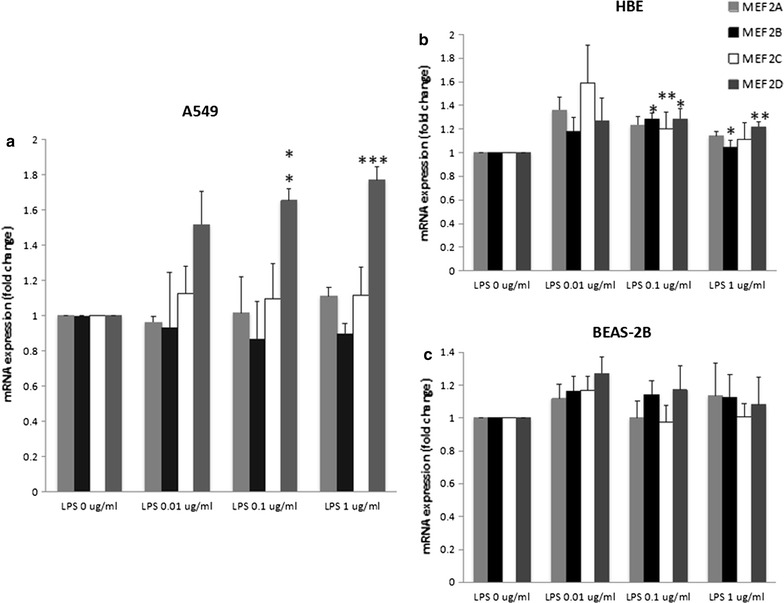
mRNA expression of MEF2s in different LPS-activated lung cell lines. **a** qRT-PCR analysis of MEF2s expression in total mRNA harvested from A549 cells challenged with PBS (LPS 0 μg/ml) or LPS at 0.01, 0.1, 1 μg/ml for 4 h; **b** qRT-PCR analysis of MEF2s expression in total mRNA harvested from HBE cells challenged with PBS (LPS 0 μg/ml) or LPS at 0.01, 0.1, 1 μg/ml for 4 h; **c** qRT-PCR analysis of MEF2s expression in total mRNA harvested from BEAS-2B cells challenged with PBS (LPS 0 μg/ml) or LPS at 0.01, 0.1, 1 μg/ml for 4 h. Data were presented as mean ± S.E.M and each group had at least six measurements (*p < 0.05, **p < 0.01, ***p < 0.001, as compared with control group)

We investigated MEF2s gene expression in different lung cell lines through qRT-PCR analysis. We found that LPS challenge significantly up-regulated the mRNA levels of MEF2D in NSCLC cell line A549 cells, as compared with those stimulated with vehicle, in accordance with the RNA-seq results (Fig. 3a). The mRNA levels of MEF2D were also up-regulated in HBE cells after LPS stimulation, but the fold changes were lower than those in LPS-activated A549 cells (Fig. 3b). As shown in Fig. 3c, levels of MEF2D were not significantly altered in BEAS-2B cells.

### MEF2D expressions increased associated with the severity of inflammation

**Fig. 4:**
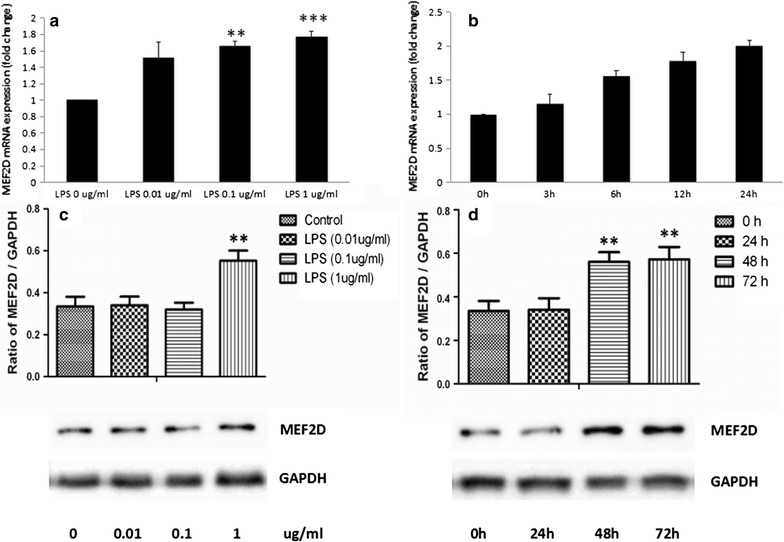
Expressions of MEF2D in LPS-activated NSCLC cell line. **a** qRT-PCR analysis of MEF2D mRNA expression in total mRNA harvested from A549 cells challenged with PBS (LPS 0 μg/ml) or LPS at 0.01, 0.1, 1 μg/ml for 4 h; **b** qRT-PCR analysis of MEF2D mRNA expression in total mRNA harvested from A549 cells challenged with LPS at 1 μg/ml from 0 to 24 h; **c** Western blotting analysis of MEF2D protein expression in A549 cells challenged with PBS (LPS 0 μg/ml) or LPS at 0.01, 0.1, 1 μg/ml for 48 h; **d** Western blotting analysis of MEF2D protein expression in A549 cells challenged with LPS at 1 μg/ml from 0 to 72 h. Data were presented as mean ± S.E.M and each group had at least six measurements (**p < 0.01, ***p < 0.001, as compared with control group)

The mRNA expressions of MEF2D in LPS-stimulated A549 cells increased in a concentration-dependent pattern and reached to the highest level when LPS concentration was 1 μg/ml (Fig. 4a, p < 0.001). LPS-stimulated expression of MEF2D mRNA increased in a time-dependent pattern as well, and reached to the highest level at 24 h after LPS stimulation (Fig. 4b, p < 0.001). The protein expressions of MEF2D also increased in a concentration- or time-dependent pattern after LPS stimulation, and reached to the highest level when LPS concentration was 1 μg/ml (Fig. 4c, p < 0.01) or at 72 h after LPS stimulation (Fig. 4d, p < 0.01), respectively, in accordance with the mRNA expressions.

### MEF2D-deficiency impaired NSCLC cell bio-behaviors

**Fig. 5:**
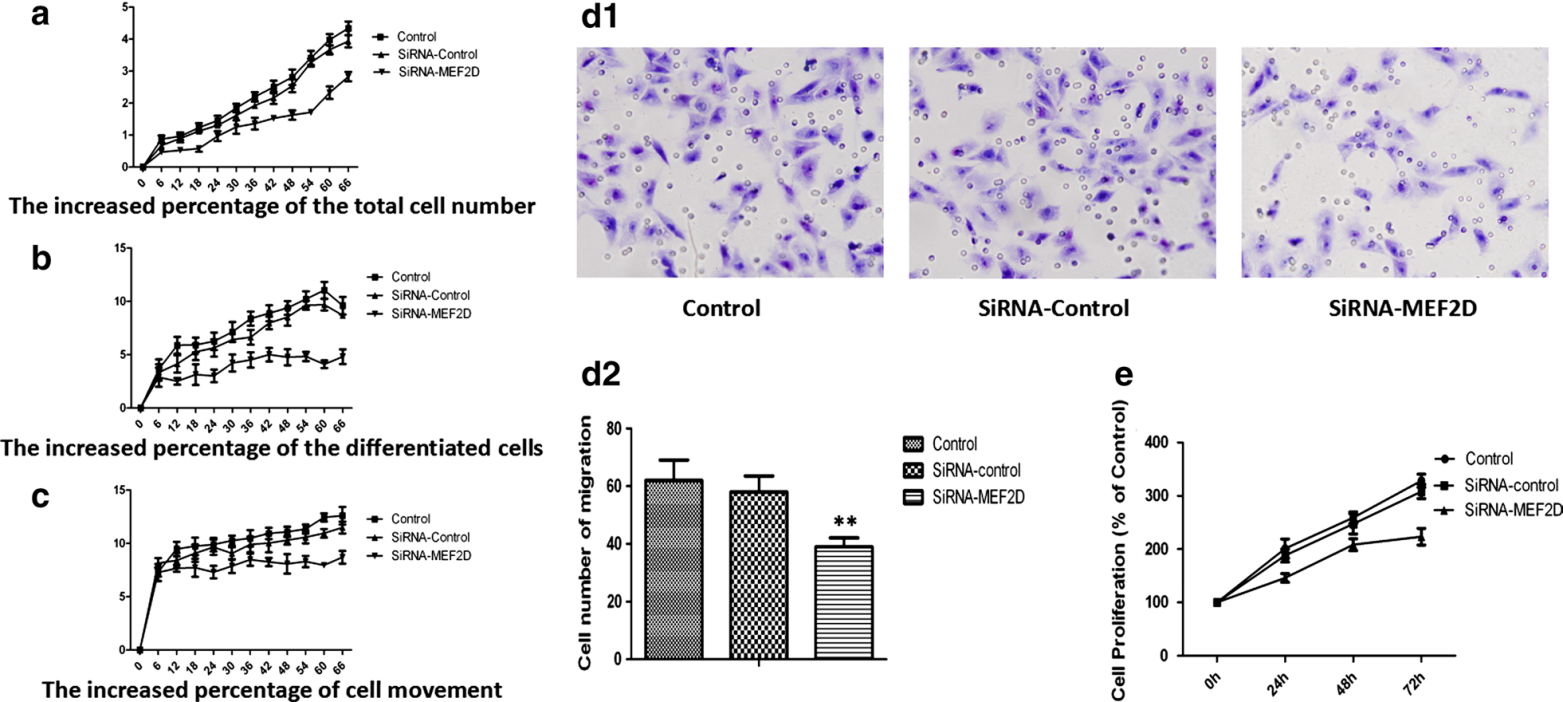
Interference of MEF2D expression impaired cell bio-behaviors in cultures of NSCLC cell line. Dynamic alterations of the cells proliferation (**a**), differentiation (**b**), and movement (**c**) by Cell-IQ Alive Image Monitoring System. **d1** Effects of MEF2D on A549 migration by Transwell assay. Figures showed Giemsa stained A549 which migrated through the permeable membrane at 48 h. Migration of MEF2D-knockdown A549 by SiRNA toward 10% FBS was significantly reduced compared to the control group. **d2** Average cell numbers of migration in three identical experiments by Transwell assay. **e** Effects of MEF2D on A549 proliferation by cell proliferation assay. Data were presented as mean ± S.E.M. of three independent experiments. **p < 0.01, as compared with control group

We used the real-time cell monitoring system to investigate the effect of MEF2D on cell bio-behaviors. We found that MEF2D-deficiency significantly reduced the capacity of cell proliferation (Fig. 5a), differentiation (Fig. 5b) or movement (Fig. 5c) in NSCLC cell line A549 cells, compared with the control cells. The role of MEF2D in A549 cells migration was further investigated by Transwell assay. As shown in Fig. 5d1, d2, MEF2D-knockdown A549 cells by siRNA had significantly impaired capacity of migration toward 10% FBS, compared with the control cells (p < 0.01). The role of MEF2D in A549 cells proliferation was further investigated by CCK-8 assay. As shown in Fig. 5e, capacity of cell proliferation was significantly reduced in MEF2D-knockdown A549 cells, compared with the control cells.

### Extracellular signal-regulated protein kinase (ERK) inhibition reversed up-regulation of MEF2D in inflammation-activated NSCLC cell line

**Fig. 6:**
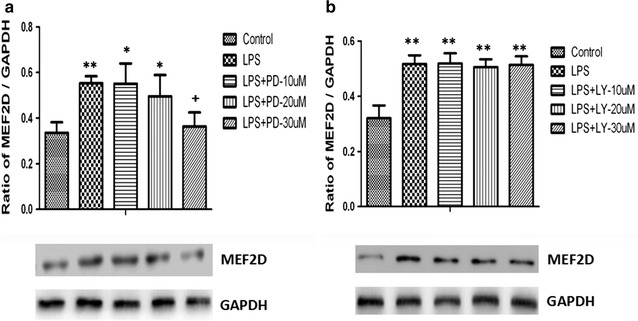
Effects of signaling pathway inhibitors on LPS-induced MEF2D expression. **a** Western blotting analysis of MEF2D protein expression in A549 cells pretreated with ERK inhibitor PD98059 at doses of 10, 20 and 30 μM for 2 h and then stimulated with LPS (1 μg/ml) for 48 h; **b** Western blotting analysis of MEF2D protein expression in A549 cells pretreated with PI3K inhibitor LY290042 at doses of 10, 20 and 30 μM for 2 h and then stimulated with LPS (1 μg/ml) for 48 h. Data were presented as mean ± S.E.M and each group had at least six measurements. *p < 0.05, **p < 0.01, as compared with control group. ^+^p < 0.05, as compared with LPS group

A549 cells respectively pretreated with ERK inhibitor PD98059 or PI3 K inhibitor LY290042 at doses of 10, 20 and 30 μM for 2 h were then challenged with vehicle or LPS at the concentration of 1 μg/ml for 48 h. Treatment with PD98509 significantly reversed LPS-induced up-regulation of MEF2D (Fig. 6a, p < 0.05), while treatment with LY290042 did not alter the LPS-induced MEF2D expression significantly (Fig. 6b).

## Discussion

MEF2D, a transcription factor of the MEF2 family, is not only critical for myogenesis, but also plays an important role in regulating diverse developmental programs [13]. It has been implicated that MEF2D is involved in different types of malignancies, including acute lymphoblastic leukemia, hepatocellular carcinoma and osteosarcoma [14–18]. Recently, MEF2D has been reported to promote the growth of lung cancer [19]. In this study, we found that MEF2D was overexpressed in clinical NSCLC tissues. Further data mining showed significantly higher MEF2D level in COPD patients with NSCLC than that in smokers, patients with COPD and no NSCLC, or patients with NSCLC and no COPD. Growing evidence has shown that patients with chronic respiratory conditions such as COPD exhibit a significantly higher risk of lung cancer [8]. It is reported that 50–90% lung cancer patients have coexisting COPD [20]. Chronic inflammation associated with COPD seems to be a critical player in lung cancer development and prognosis [21, 22], related to 15–20% of cancer deaths [23], while little is known about the underlying mechanism. The results prompted us to further speculate whether MEF2D might act as a communicator between inflammation and lung cancer.

The present study adopted an in vitro model based on LPS-stimulated A549 to explore the underlying link between inflammation and lung cancer. Our data showed that MEF2D expression was significantly elevated in inflammation-activated NSCLC cell line A549 through Ion Torrent-based RNA-sequencing analysis, as were genes associated with MEF2D network. LPS challenge specifically up-regulated the MEF2D mRNA expression in A549 significantly rather than in bronchial epithelial cell lines HBE or BEAS-2B cells. Our data also demonstrated that LPS challenge up-regulated the mRNA or protein levels of MEF2D in A549 in a LPS-concentration dependent and time dependent manner. It seems that the influencing roles of inflammation in the expression of MEF2D are related with the severities.

Diverse calcium-dependent signaling pathways have been revealed as regulators of MEF2 activity, including calcium/calmodulin-dependent protein kinase (CaMK) signaling, calcineurin signaling, and mitogen-activated protein kinase (MAPK) signaling [10]. MAPKs are mainly divided into three signaling cascades: ERK, p38 kinase and c-jun N-terminal kinase (JNK) pathways. In accordance with the previous study [24], we found that the ERK signaling pathway may play the critical and dependent role in the mechanism of MEF2D production of A549, evidenced by the finding that the over-production of MEF2D by LPS-challenge was prevented by ERK inhibitor. Further study is needed to demonstrate MEF2D targeted genes after inflammation challenge.

Inflammation plays multifaceted roles in all stages of tumorigenesis, including malignant transformation, tumor initiation to invasion, and metastasis of established tumor [25]. Cancer-related inflammatory microenvironment, mainly composed of inflammatory cells and mediators, has been accepted as a significant factor through which inflammation contributes to the multiple capabilities of cancer [26]. Our previous study reported that inflammation-activated lung cells could act as the initiators and/or secondary sources of the development of cancer microenvironment [27]. Consistent with our previous study, we provide further evidence that inflammatory conditions might contribute to remodeling of the cancer microenvironment through up-regulating MEF2D expression.

During a host response to bacterial pathogens, activation of MEF2 by p38 kinase pathway has been related to expression of pro-inflammatory cytokines [28]. In primary human T lymphocytes, MEF2D is required for the synthesis of IL-2 mRNA and secretion of IL-2 in response to T cell receptor stimulation [29]. MEF2 participates in the regulation of B lymphocyte proliferation and survival after B cell receptor stimulation [30]. MEF2D is identified as an important regulator of IL-10 gene expression [31]. Given the critical roles of MEF2 in regulation of inflammatory responses, up-regulation of MEF2D might lead to remodeling of cancer microenvironment through lymphocytes recruitment or mediators production during inflammatory condition. Further in vitro study is needed to clarify the underlying mechanism.

MEF2D has been reported to serve as oncogene in B cell acute lymphoblastic leukemia [15] and hepatocellular carcinoma [16, 32–34] through promotion of colony formation and proliferation, inhibition of apoptosis, epithelial-mesenchymal transition and invasiveness. MEF2D also acts as tumor suppressor in liposarcoma, leiomyosarcoma [35] and rhabdomyosarcoma [36, 37] through promotion of cell proliferation and anchorage independent growth and inhibition of differentiation. Consistent with the previous studies, we found that MEF2D played a critical role in bio-behaviors of NSCLC cells. While targeted knockdown of MEF2D gene via siRNA significantly impaired the capacity of cancer cell differentiation, proliferation or movement. Up-regulation of MEF2D during inflammatory condition might favor lung cancer cell differentiation, proliferation, or movement.

Briefly, inflammatory conditions led to increased MEF2D expression, which might further contribute to the development of malignancy through influencing cancer microenvironment and cell bio-behaviors. As far as we know, this is the first study focused on MEF2D as a “communicator” between chronic inflammation and lung cancer. MEF2D might be a potential biomarker for patients with chronic respiratory diseases, such as COPD, who are at risk of developing lung cancer.

However, the present study has some limits. Although we adopted an in vitro model based on LPS-stimulated A549 to mimic inflammatory conditions of airways, there are still differences between chronic airway inflammation in vivo and the in vitro model. Much more remains to be learned about the regulatory mechanism of MEF2D to communicate between chronic inflammation and lung cancer.

## Conclusions

Taken together, the present study demonstrated significantly higher MEF2D level in COPD patients with NSCLC than that in smokers, patients with COPD and no NSCLC, or patients with NSCLC and no COPD. Inflammation challenge increased MEF2D expression in A549, associated with the severity of inflammation, while ERK inhibition could reverse the up-regulation of MEF2D. MEF2D played a critical role in NSCLC cell differentiation, proliferation, or movement. MEF2D might contribute to the development of lung cancer through influencing cancer microenvironment and cell bio-behaviors during inflammatory conditions. MEF2D provides a cross-talk between chronic inflammation and lung caner. MEF2D could be a potential biomarker predicting risk of lung cancer among patient with chronic respiratory diseases. More studies are still needed to explore the further mechanism between chronic inflammation and lung cancer.

## Additional file

**Additional file 1: Table S1.** Sequences of siRNA targeting MEF2D.

## Abbreviations

COPD: chronic obstructive pulmonary disease
qRT-PCR: quantitative real time polymerase chain reaction
GEO: Gene Expression Omnibus
MEF2D: myocyte enhancer factor 2D
NSCLC: non-small-cell lung carcinoma
LCC: large-cell carcinoma
SCLC: small-cell lung carcinoma
MEF2: myocyte enhancer factor 2
ERK: extracellular signal-regulated protein kinase
siRNA: small interfering RNA
CaMK: calcium/calmodulin-dependent protein kinase
MAPK: mitogen-activated protein kinase
JNK: c-jun N-terminal kinase
