# Supplementary material for: Myocyte enhancer factor 2D provides a cross-talk between chronic inflammation and lung cancer
Source: J Transl Med. 2017 Mar 24;15:65. doi: 10.1186/s12967-017-1168-x (PMC5366127; doi:10.1186/s12967-017-1168-x)
Supplement: Supplementary file 1 — Additional file 1: Table S1. Sequences of siRNA targeting MEF2D. [file 12967_2017_1168_MOESM1_ESM.docx]

**Table S1.** Sequences of siRNA targeting MEF2D.

Three different sequences of siRNA targeting MEF2D are as follows:

| MEF2D  (Gene ID: 4209) | Sequences |
| --- | --- |
| S1 | GCTTGATACCTGGACATTAAA |
| S2 | GCCTGATGAAGAAGGCGTATG |
| S3 | GCGAGATCGCACTCATCATCT |

After optimizing the efficacy of transfection, we used “S3” to perform the small interfering RNA transfections.
